# Full Likelihood Analysis of Genetic Risk with Variable Age at Onset Disease—Combining Population-Based Registry Data and Demographic Information

**DOI:** 10.1371/journal.pone.0006836

**Published:** 2009-08-31

**Authors:** Janne Pitkäniemi, Sirkka-Liisa Varvio, Jukka Corander, Nella Lehti, Jukka Partanen, Eva Tuomilehto-Wolf, Jaakko Tuomilehto, Andrew Thomas, Elja Arjas

**Affiliations:** 1 Department of Public Health, University of Helsinki, Helsinki, Finland; 2 Department of Mathematics and Statistics, University of Helsinki, Helsinki, Finland; 3 National Public Health Institute, Department of Health Promotion and Chronic Diseases Prevention, Helsinki, Finland; 4 Finnish Red Cross Blood Service, Department of Tissue Typing, Helsinki, Finland; Ohio State University Medical Center, United States of America

## Abstract

**Background:**

In genetic studies of rare complex diseases it is common to ascertain familial data from population based registries through all incident cases diagnosed during a pre-defined enrollment period. Such an ascertainment procedure is typically taken into account in the statistical analysis of the familial data by constructing either a retrospective or prospective likelihood expression, which conditions on the ascertainment event. Both of these approaches lead to a substantial loss of valuable data.

**Methodology and Findings:**

Here we consider instead the possibilities provided by a Bayesian approach to risk analysis, which also incorporates the ascertainment procedure and reference information concerning the genetic composition of the target population to the considered statistical model. Furthermore, the proposed Bayesian hierarchical survival model does not require the considered genotype or haplotype effects be expressed as functions of corresponding allelic effects. Our modeling strategy is illustrated by a risk analysis of type 1 diabetes mellitus (T1D) in the Finnish population-based on the HLA-A, HLA-B and DRB1 human leucocyte antigen (HLA) information available for both ascertained sibships and a large number of unrelated individuals from the Finnish bone marrow donor registry. The heterozygous genotype DR3/DR4 at the DRB1 locus was associated with the lowest predictive probability of T1D free survival to the age of 15, the estimate being 0.936 (0.926; 0.945 95% credible interval) compared to the average population T1D free survival probability of 0.995.

**Significance:**

The proposed statistical method can be modified to other population-based family data ascertained from a disease registry provided that the ascertainment process is well documented, and that external information concerning the sizes of birth cohorts and a suitable reference sample are available. We confirm the earlier findings from the same data concerning the HLA-DR3/4 related risks for T1D, and also provide here estimated predictive probabilities of disease free survival as a function of age.

## Introduction

Family data utilized in genetic association studies of rare diseases are usually ascertained by initially recruiting individuals with the phenotype of interest from some background population. After this initial study phase, it is possible to gain information about the relatives of the recruited subjects. Such an ascertainment procedure is usually taken into account in the statistical analysis of familial data by constructing either a retrospective or prospective likelihood expression, which conditions on the ascertainment event [1]. Complex ascertainment procedures often lead to inferential difficulties; recently proposed computationally intensive methods can however provide ways to resolve such issues [2].

In the statistical analysis of variable age at onset diseases, the versatility of traditional survival analysis methods has been frequently demonstrated in genetic linkage and association studies [3]–[7]. Recent advances in modern non-parametric Bayesian survival modeling have however mainly been utilized outside the domain of genetic research [8]–[9]. To create a likelihood-based framework for estimating disease risks associated with the genetic information and other possible factors available, we use here an approach where a population based ascertainment procedure is combined through a statistical model with the demographic data describing also the non-ascertained part of the target population.

Our framework is illustrated by a model of the T1D risks associated with polymorphic markers located in the HLA region of chromosome 6 in the Finnish population. Although our approach is more generally applicable, the model framework is presented directly in the T1D context in order to make it more easily accessible for readers with an interest in genetic epidemiology rather than in statistical methodology per se. The family based T1D data set was collected as a part of the DiMe study [10], and has been previously analyzed by other statistical methods [11]–[12]. The additional reference data utilized in the present work are taken from a large sample (∼20,000 individuals) of unaffected Finns at the Finnish Bone Marrow Donor Registry (BMDR), who had been serotyped for the same HLA loci as the family members included in the DiMe Study. These two sources of information are further appended with the available demographic facts about the population at risk during the ascertainment period. Since the dominance effects of HLA-DRB1 are known to be highly genotype dependent, we chose to model the effects of HLA-DRB1 genotypes, rather than alleles [13], as has been done in the previous analyses using these same data. All genotype-associated risks are here estimated jointly within a hierarchical Bayesian hazard modeling framework. Similarly, in our risk model it is not assumed that the considered haplotype effects could be expressed as corresponding functions of allele effects.

In the next section we provide some details of the available data sets, introduce a risk model for the genotype/haplotype effects on age at the onset of the disease, and derive the corresponding likelihood function and the joint posterior density of all model parameters. Then the numerical results from the analysis are presented. In the final part of the paper, we discuss some merits of the proposed method for accounting for the effect of the ascertainment, evaluate the empirical results, and suggest some possibilities for future work in this area.

## Materials and Methods

### Data sources

We consider the situation in which ascertainment is based on all incident cases in the target population during the enrollment period (the recruitment window). This then leads to observed data on sibships with a proband, and his/her siblings who have been at risk for the disease under investigation. In our application, the observed data consist of four parts:

1. Age at (possible) onset of T1D among all members of the ascertained families, who have been at risk during the follow-up period. Here, only diagnoses reported before the age of 15 years are considered. A total of 768 families are included in the recruited group.
2. HLA genotypes of the members of the ascertained nuclear families.
3. The numbers of the individuals in the background population at risk, comprising of the individuals who were alive during the calendar period included in the recruitment window (*y = *1987, 1988, 1989) and belonging to the age groups *a* = 0,…,14.
4. The HLA genotypes from approximately 20,000 unrelated, healthy Finns. This population reference group thus corresponds to the control individuals utilized in case-control association studies.

**Figure 1:**
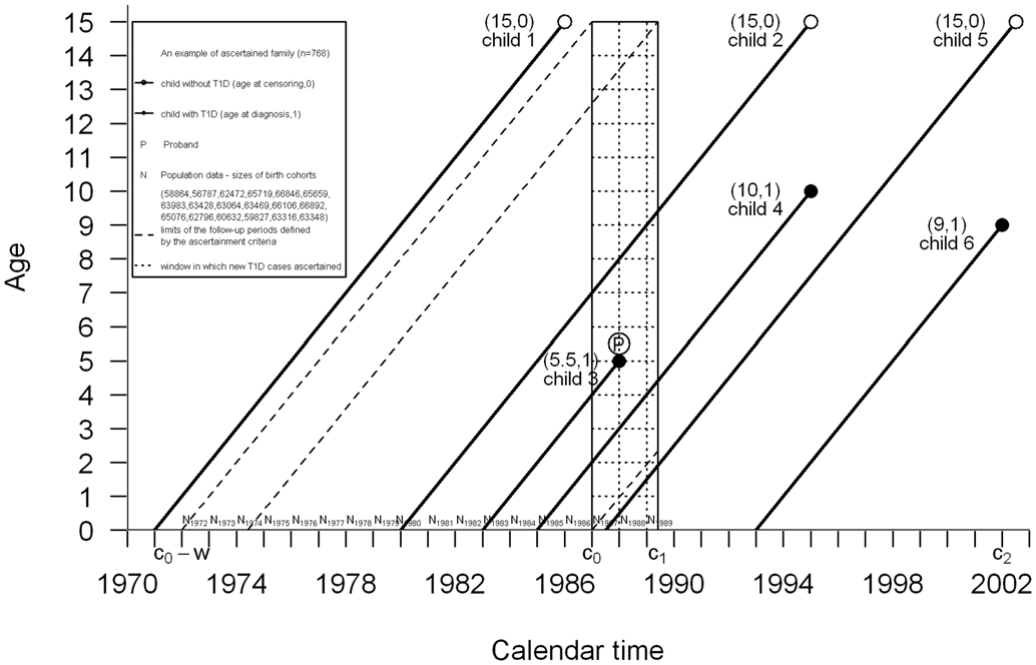
Lexis diagram of the population-based ascertainment of Finnish sibships with Type 1 diabetes. An example of an ascertained sibship included in the population-based disease registry data, because an individual (proband) younger than 15 years who was diagnosed with T1D during the recruitment period 1.1.1987–30.4.1989.

The Lexis diagram displayed in Figure 1 illustrates the ascertainment procedure for sibships and the data needed to construct the likelihood for an absolute risk model. Let *i* = 1, …, *I* index all the ascertained families, *j* = 1, …, *J_i_* indexing the individuals (siblings including the proband) in the *i*th family, and *δ_ij_* being the indicator for right censoring of the follow-up (*δ_ij_* = 1 if individual *j* in family *i* was diagnosed with T1D in the recruitment window, and *δ_ij_* = 0 if right censored. Let *X_ij_* represent the age at onset or the age at a censoring event. Further, for the siblings in the family data, let 
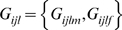
 be the phase unknown marker alleles over the set of loci of interest, where *l* = 1,…, *N_L_*, and let 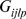 be phased alleles where *p = m, f* index the parents of the *i*th family. The alleles received from the mother (*m*) and the father (*f*) are indexed accordingly, and an analogous indexing is later used for haplotypes as well. Parental genotypes are denoted by 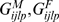. In order to actually model risks associated with haplotypes, it is assumed that the haplotype phases have been resolved computationally prior to the risk analysis. The HLA-A, HLA-B and HLA-DRB1 loci are included in the current data of the HLA region. The observed data of the ascertained sibships (Parts 1 and 2) are thus collectively represented by the set 
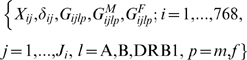



The T1D families were collected from the nationwide T1D registry in Finland, ascertained within the DiMe study [10] through a child under the age of 15 years and diagnosed between January 1, 1987 and April 30, 1989. The Childhood Diabetes in Finland (DiMe) study was a large population-based genetic-epidemiologic family study of T1D. Nationwide, all T1D cases under the age of 15 in Finland were diagnosed. The cut-off age was chosen purely for practical reasons. Newly diagnosed children under the age of 15 years with T1D were hospitalized in the pediatric wards in Finland and therefore were easier to recruit than older subjects with T1D. T1D status was checked against the data of the National Drug Registry. Of the 801 cases in the study 800 had also been registered in the Drug Registry and one person died soon after the diagnosis. The participation rate in the study was approximately 95%. Parents and siblings of the 801 probands were also asked to participate. Extensive questionnaires were filled in and blood samples were taken from participants. Probands, their parents and siblings were HLA genotyped at A, B, C and DR loci using conventional serology (768 families). Details of study procedures, especially data collection, are described elsewhere [10]. For the ascertained families we constructed a follow-up of T1D until 31.12.2001, by updating the T1D status and the date of diagnosis from the National Hospital Discharge Registry (Part 1). An individual was thus considered censored if he/she reached the age of 15 without having been diagnosed to have T1D, or was less than 15 years old and was not yet diagnosed on 31.12.2001.

In summary, we have for the comprehensive statistical analysis of HLA-A, HLA-B, and DRB1 loci, a total of 768 ascertained families comprising 1,944 probands or siblings. HLA genotypes were available from 1,342 probands or siblings (684 families, see Table S1). The DiMe study protocol has been described in detail elsewhere [14] and it has been approved by the ethics committees of the participating hospitals. Informed consent was obtained from the families taking part in the study. The HLA genotyping (Part 2) of these families was done in the National Public Health Institute Laboratory using classical serology. Haplotypes within the sibships in the ascertained family data were established using the SimWalk2 software [15], based on the available information about the parental HLA genotypes.

In order to include demographic information in the likelihood (Part 3), we assume that the sizes of the birth cohorts are known for the relevant time period. Here they are denoted by 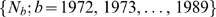 (see Figure 1). Let the starting and end points of the recruitment window be denoted by *c*_0_ and *c*_1_, respectively, and let *w* be the maximum age at which a subject may be ascertained (here less than 15 years). As the ascertainment is population-based, with a negligible magnitude of missing cases during the recruitment period, it is possible to incorporate in the risk model information about all individuals who were born during a certain calendar time interval and who had passed the recruitment window without being ascertained. This corresponds to all subjects born in the population between the calendar time points *c*_0_ *-w* and *c*_1_. We divide subjects born in the general population such that they could have become probands but did not, into three sets according to the recruitment window as shown in Fig. 1. Subjects born during the time interval (*c*_0_-*w*, *c*_1_-*w*) are in the sequel indexed by *k*_1_, respectively subjects born during (*c*_1_-*w*, *c*_0_) are in the sequel indexed by *k*_2_, and finally, subjects born during the recruitment period (*c*_0_, *c*_1_) are indexed by *k*_3_. The demographic information allows us to treat these individuals systematically in the risk model via the known sizes of the birth cohorts.

The genetic information concerning HLA for the demographic data (*N_b_*) is in our model formulation inferred from the genotypic data in the population reference sample (Part 4). From the BMDR we obtained comparable genotypes for HLA-A, HLA-B and DRB1 loci for 19,386 unrelated individuals. The BMDR database includes healthy individuals who had agreed to volunteer for possible bone marrow donation. The Finnish BMDR registry is owned by the Finnish Red Cross Blood Service and it is not a public database. The primary purpose of this registry is to search for potential bone marrow donors for transplantations. All individuals who have joined the BMDR registry have given their written consent for anonymous registry based research. Anybody full filling the health criteria (roughly equivalent to those required for blood donation) and willing to donate stem cells (or “bone marrow”) for stem cell transplantation can join the registry. The registry does not accept joining that is based on, or motivated, by ‘targeted’ donation to e.g. relative or friend only, but the registree must be willing to donate to any patient. The bone marrow transplantations between related individuals are handled separately from the BMDR. Hence, we had no reason to believe that members of the BMDR are more related to each other than general population. From subjects who had given written consent, about 10 mL of peripheral blood was drawn for standard HLA typing. HLA typing included the standard serological HLA-A and -B typings and either serological (early samples) or DNA-based DRB1 typing. Genotype consistent HLA haplotypes for the BMDR data were constructed with the PHASE software [16]. In order to be able to compare HLA genotypes/haplotypes between different data sources or typing methods, all HLA types were converted to pooled alleles (see supplementary material), if necessary, to the serological main specificities according to the official HLA nomenclature [17]. Let 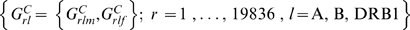 be the set of genotypes for the unrelated individuals in the BMDR database, using a notation analogous to the familial data. These reference individuals are known *not* to have acquired T1D before age *w*. The genotype and haplotype frequencies for this reference population are collectively denoted by 
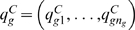
 and 
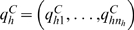
, respectively.

### Modeling of genetic risks and of the family-based ascertainment procedure

#### Hazard model

Establishing associations between T1D and the classical highly polymorphic linked marker loci within the HLA region is a challenging task from a statistical perspective, due to the large number of parameters in the risk model formulation that need to be estimated. The first part of the risk model specifies the hazard of acquiring T1D for individual *j* in family *i*, as a function of age *a*. For both the genotype and haplotype effect models we use a discrete time hazard model and index age by *a* = 0,…,14 corresponding to the age intervals 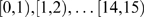. For the genotype effect model, the hazard is assumed to be of form
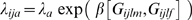
(1)


Here *λ_a_* is a baseline hazard in the population and *β*[*G_ijlm_*,*G_ijlf_*] are the genotype effects representing the molecular marker information at *l* = HLA-A, HLA-B, HLA-DRB1, each locus being considered and analyzed separately. For the analysis of the effects of the HLA- A, -B and DRB1 haplotypes, we use the corresponding “marginal” model in which the effect is always randomly attributed to one of the two haplotypes 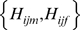 of a considered individual. More precisely, we consider effects of the form *β* [*H_ijp_*], *p = m, f*, where the index *p*, or phase, is treated as missing data in the corresponding Bayesian model, assigning the prior probability of 0.5 to both *m* and *f*.

### Likelihood expression

An assumption of the conditional independence of individual disease onset times, conditionally given the model parameters, leads to the following likelihood expression for the combined set of data: 
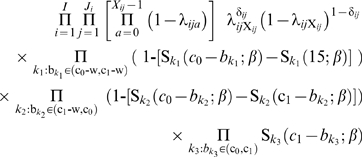
(2)


The first factor in this likelihood expression is the direct contribution of the DiMe family data, with *i* = 1, …, *I* indexing all the ascertained families, *j* = 1, …, *J_i_* indexing the individuals (siblings including the proband) in the *i*th family, and *δ_ij_* being the indicator for right censoring of the follow-up (*δ_ij_* = 1 if individual *j* in family *i* was diagnosed with T1D in the recruitment window, and *δ_ij_* = 0 if right censored, cf. Figure 1).

The next three factors in (2) are the contributions of the individuals, indexed here with *k*_1_, *k*_2_ and *k*_3_, in the background population. These indexes represent the non-ascertained individuals, who could have been diagnosed with T1D in the “ascertainment window”, but who were in fact not ascertained to the DiMe sample. These non-ascertained individuals are considered individually in the “full likelihood” function (2). Let 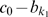 be the age of individual *k*_1_, who was born at 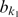, at the beginning of the recruitment period. Similarly 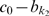 and 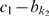 are the ages of an individual *k*_2_ born at 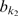
at the beginning and at the end of the recruitment interval, and 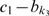
is the age of an individual born at 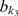 at the end of the recruitment interval. Then 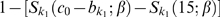, 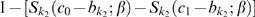 and 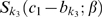 are the corresponding probabilities of not being diagnosed with T1D in that interval, expressed here as a function of the parameters *β* of the hazard model (1).

Note that since the hazard model (1) also contains in its arguments the genotype/haplotype of the considered individual, which is unknown for individuals of types *k*_1_, *k*_2_ and *k*_3_, they need to be integrated away from the corresponding expressions of the survival function. For the distribution of the genotypes/haplotypes we made, for computational reasons, a shortcut and used their empirical frequencies in the BMDR data base as the prior. (A theoretically more satisfying solution could have been to postulate the Dirichlet(1,…,1) prior for the frequencies and then update their estimates by sampling, concurrently with the estimation of other model parameters and using the BMDR genotypes/haplotypes as data. However, in view of the size of this data base we thought that doing so would not be worth the extra effort). Note also that, with the genotype/haplotype information being integrated away, all individuals of type *k*_1_ born at the same time 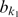
are treated as exchangeable, and therefore the corresponding product in (2) becomes a power, where the exponent is the number of individuals (excluding those belonging to the DiMe families) born at 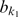. These numbers are obtained directly from the demographic data.

### Bayesian inference

Next, we describe how the likelihood (2) can be calculated and how the resulting parameter estimates are obtained. Applying the Bayesian approach to statistical inference, the likelihood is complemented with the joint prior distributions of all model parameters and latent variables of interest. Here this is done by following the principles of hierarchical Bayesian modeling and by specifying the distributions appearing on the right hand side of the following expression
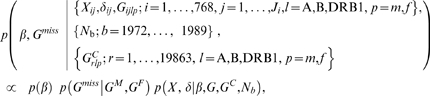
(3)in the OpenBugs MCMC software [18], where *G^miss^* are the missing HLA genotypes/haplotypes in the ascertained sibships. The prior distributions used for the model parameters *β* and missing HLA genotypes were specified as follows. The age specific log baseline hazards were assigned independent Gaussian priors according to log(*λ_a_*) with mean -8 and precision (reciprocal of variance) equal to 0.000001, where *a* = 0,…,14. The genotype/haplotype effects were assigned independent truncated normal (−10,10) prior distributions with mean 0.0 and precision 0.000001. This is, effectively, the uniform distribution over the interval (−10, 10). Particularly when noting that *β* is in the hazard model (1) in the exponent, this interval can be said to cover more than adequately all plausible values of genotype or haplotype effect.

The prior of the missing HLA genotypes 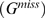 in the family-based data was treated according to available parental HLA genotype information. Two distinct situations of missing HLA genotypes can be identified among the ascertained sibships:

1. Both parents were genotyped, but not all children. Here Mendel's law of segregation was utilized to assign the two parental alleles with equal probabilities, and then applying the method of data augmentation, as a part of the resulting Bayesian computation.
2. Genotype information was missing from at least one parent and no children were genotyped. Here, the logically consistent way to treat the missing data problem would involve using Bayes' formula, with the haplotype frequencies of the parents of the ascertained families as a “prior” probability distribution, while Mendel's law defines the likelihood. However, in the 40 ascertained families, where parental HLA information was missing and some sibs were genotyped, the inference was implemented as in 1), despite the fact that some inconsistent genotypes may then be obtained. This was done in order to avoid a further increase in the computational burden of implementing the already complex model when using OpenBugs software [18].

In the final implementation we first obtained the posterior mean of the baseline hazard for T1D using a model without the HLA effects. In the reported analyses of the HLA genotype and haplotype effects, the baseline hazard was held fixed at this posterior mean. This strategy was chosen in view of the fact that the Finnish birth cohorts, approximately 60,000 each and effectively forming the risk sets of our population based analysis, are so large that the joint estimation of the baseline hazards with the other model parameters would have hardly led to numerically different estimates. The reported analyses are based on 5,000 iterations (5,000 burn-in), and the estimates of genotype effects passed the convergence diagnostics criteria of Geweke [19] (R-program package CODA). In order to describe the age dependency in the HLA genotypes, we calculated the genotype and haplotype specific predictive disease free survival functions and its' 95% credible intervals. It is the expectation of disease free survival function, with respect to the joint posterior distribution of all model parameters [20].

## Results

**Figure 2:**
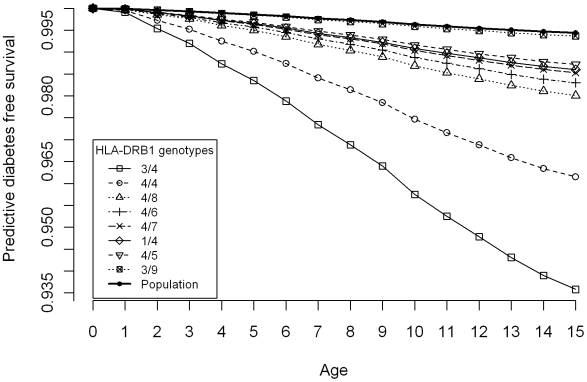
Predictive disease free survival of T1D for some high risk HLA DRB1 genotypes.

**Figure 3:**
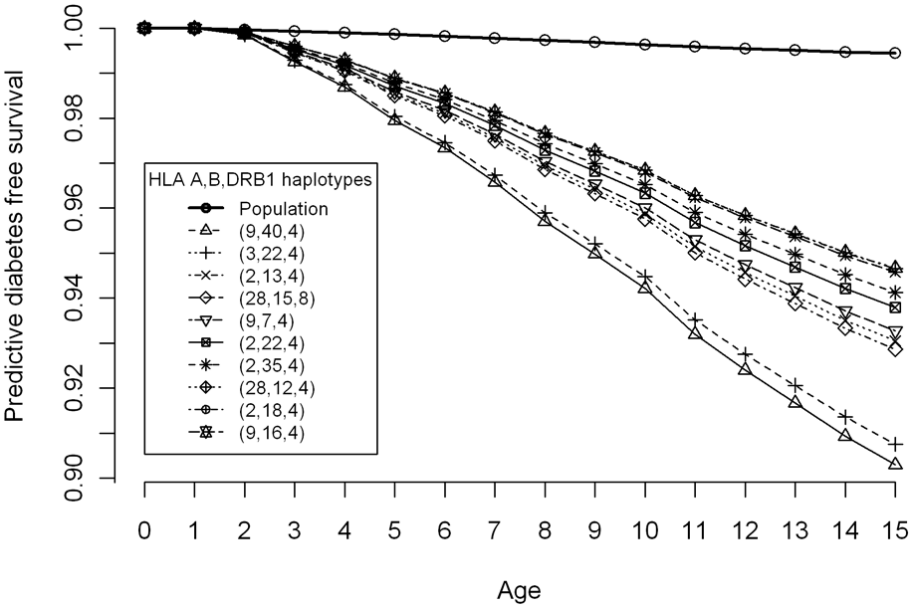
Predictive disease free survival of T1D for some high risk HLA A,B and DRB1 haplotypes.

Given the complexity of the genetic information, only concise summaries of the results can be reported here. The numbers and percentages of particular genotype/haplotype carriers with T1D, as well as the corresponding healthy carriers in the ascertained sibships and in the BMDR sample, together with the associated predictive probabilities of T1D free survival, are given in Table 1. Predictive probabilities of T1D free survival are shown in Figure 2 for some high risk HLA DRB1 genotypes and in Figure 3 for a set of selected haplotypes.

**Table 1:** Numbers of genotype/haplotype carriers and predictive disease free survivals (95% credible intervals) of certain HLA-A, B and DRB1 genotypes/haplotypes§.

HLA Genotype/Haplotype	# of cases	% of cases	# of healthy carries in ascertained sibships	% of healthy carries in the ascertained sibships	Number of healthy carriers in the BMDR	Predictive T1DM free survival before age 5 (95% credible interval)	Predictive T1DM free survival before age 10 (95% credible interval)	Predictive T1DM free survival before age 15 (95% credible interval)
DRB1								
3/4	173	23.44	42	6.84	481	0.984 (0.981;0.986)	0.958 (0.951;0.964)	0.936 (0.926;0.945)
4/4	88	11.92	42	6.84	417	0.990 (0.988;0.992)	0.975 (0.969;0.980)	0.962 (0.954;0.969)
4/8	71	9.62	50	8.14	561	0.995 (0.994;0.996)	0.987 (0.984;0.990)	0.980 (0.975;0.984)
…								
3/3	8	1.08	6	0.98	231	0.999 (0.998;0.999)	0.996 (0.994;0.998)	0.994 (0.990;0.998)
…								
1/2	2	0.27	15	2.44	1104	1.000 (1.000;1.000)	1.000 (1.000;1.000)	1.000 (0.999;1.000)
2/6	0	0	8	1.3	862	1.000 (1.000;1.000)	1.000 (1.000;1.000)	1.000 (1.000;1.000)
B								
8/56	21	2.85	7	1.14	88	0.992 (0.988;0.995)	0.976 (0.964;0.985)	0.962 (0.944;0.976)
8/15	62	8.40	18	2.93	447	0.996 (0.994;0.997)	0.987 (0.983;0.990)	0.979 (0.974;0.984)
…								
7/17	7	0.95	16	2.61	744	1.000 (1.000;1.000)	1.000 (0.999;1.000)	1.000 (0.998;1.000)
5/7	2	0.27	6	0.98	266	1.000 (0.999;1.000)	0.999 (0.998;1.000)	0.999 (0.997;1.000)
A								
1/9	32	4.34	20	3.26	314	1.000 (0.999;1.000)	0.998 (0.996;1.000)	0.998 (0.994;1.000)
2/3	135	18.29	113	18.4	3292	0.999 (0.992;1.000)	0.998 (0.978;1.000)	0.996 (0.966;1.000)
A-B-DRB1								
2 22 4	80	4.71	35	1.598	306	0.983 (0.979;0.987)	0.957 (0.946;0.967)	0.935 (0.918;0.950)
2 18 4	13	0.77	4	0.183	51	0.986 (0.974;0.994)	0.963 (0.934;0.984)	0.944 (0.901;0.976)
9 16 4	54	3.18	27	1.233	228	0.985 (0.980;0.990)	0.962 (0.950;0.973)	0.943 (0.924;0.959)
9 15 4	29	1.71	5	0.228	135	0.987 (0.981;0.992)	0.967 (0.952;0.980)	0.950 (0.927;0.970)
3 35 4	33	1.94	23	1.05	199	0.990 (0.986;0.994)	0.974 (0.963;0.983)	0.961 (0.944;0.975)
…								
1 8 3	131	7.72	86	3.927	1867	0.998 (0.997;0.999)	0.995 (0.993;0.997)	0.992 (0.989;0.995)
…								
3 35 1	60	3.53	70	3.196	3242	1.000 (1.000;1.000)	1.000(0.999;1.000)	1.000 (0.999;1.000)
3 7 2	5	0.29	32	1.461	1388	1.000 (1.000;1.000)	1.000 (1.000;1.000)	1.000 (1.000;1.000)

§Pooled allele labels based on the official HLA nomenclature (http://www.anthonynolan.org.uk/HIG/).

A9 = (9,23,24), A19 = (19,29,30,31,32,33), A28 = (28,68,69), B5 = (5,51,52),

B12 = (12,44,45), B14 = (14,64,65), B15 = (15,62,63,75), B16 = (16,38,39),

B17 = (17,57,58), B56 = (22,55,56), B21 = (21,41,50), B40 = (40,60,61),

DR2 = (2,15,16), DR3 = (3,17,18), DR5 = (5,11,12), DR6 = (6,13,14).

As could be expected from many earlier studies, the heterozygous genotype DR3/DR4 at the DRB1 locus was associated with the lowest predictive probability of the T1D free survival to the age of 15, the estimate being 0.936 (0.926; 0.945 95% credible interval), compared to the average population T1D free survival probability of 0.995. The effect of DR4 homozygote was also strong with associated probability of 0.962 (0.954; 0.969) for T1D free survival. Carriers of DR1/DR2, a common genotype in the reference population and of DR2/DR6, had virtually no risk of T1DM before the age of 15, with a predictive probability for T1D free survival very close to 1. All DRB1 genotypes associated with a lower predictive probability of T1D free survival than the population average contained the DR4 allele: DR3/DR4, DR4/DR4 DR4/DR8, DR4/DR6, DR4/DR7, DR1/DR4, and DR4/DR5.

At the HLA B locus, the genotype associated with the smallest predictive probability of T1D free survival to the age of 15 was B8/B22, for which the estimate was 0.962 (0.944; 0.976). Two common B-locus genotypes, viz. B7/B35 and B12/B35, were observed to have only a few carriers among the diagnosed cases while each had more than 50 carriers in the reference sample and had therefore a predictive probability for T1D free survival that was very close to one. Of the considered HLA-A locus genotypes, A1/A9 genotype conferred the highest T1D risk, with a predictive probability of 0.998 (0.994; 1.000) for T1D free survival among all HLA-A genotypes.

Finally, the analysis of haplotypes revealed several haplotypes with non-neutral association with T1D, in the sense of having a smaller predictive probability of T1D free survival to 15 years than the population average. Of these, the three highest ranked haplotypes, with more than 50 carriers in the reference sample, were A2/B22/DR4 0.935 (0.918; 0.950), A2/B18/DR4 0.944 (0.901; 0.976) and A2/B15/DR4 0.943 (0.924; 0.959). Notably, all but one among the top ten ranked haplotypes contained the HLA DR4 allele.

## Discussion

To our knowledge, genetic risk estimation from an ascertained familial data for a variable age at onset disease has not been earlier approached by a full likelihood, or Bayesian, method utilizing demographic information. The problem of non-random ascertainment has been usually approached by formulating a conditional likelihood [21], which leads to the removal of some individuals from the study material in order to avoid an outcome-based ascertainment bias. In our approach such an exclusion procedure is not needed and all ascertained familial data can be used in the statistical analysis. The basic modeling assumption in the present work is that the putative effects associated with the observed molecular information modulate a common age-dependent baseline hazard in a multiplicative fashion. Also, we do not assume any particular functional form for the genotype or haplotype effects, i.e., they are not assumed to be decomposable into some corresponding allelic effects.

Direct comparisons of our results to previously published studies concerning genotype/haplotype risks of HLA for T1D are difficult, as our analysis is restricted to the serotype level due to restrictions in the available data, whereas the risk estimates are currently provided at a finer molecular resolution. Thus our analysis of the T1D data should be viewed primarily as an example illustrating the potential of the likelihood based approach. The T1D data from the DiMe Study have been analyzed earlier for the allelic, genotypic, and/or haplotypic relative risks, by estimating separately the risk associated with each haplotype by the ratio of frequencies of transmitted and non-transmitted haplotypes [22], [14], or by assuming multiplicative genotype and haplotype dominance effects on TIDM relative risk [11]–[12]. The major difference in our model compared to that of Thomas et al. [12] is that we do not assume multiplicative dominance effects between alleles at the same locus but rather model directly the effects of genotypes. Despite this difference, we observe a similar ranking of the locus and haplotype effects as in Thomas's et al., with the DRB1 locus genotypes having the largest effect. Of all genotypes and common haplotypes considered, the DR3/DR4 genotype was associated with the smallest probability of surviving T1D free to the age of 15. The association between T1D and HLA-DRB1 DR3/DR4 genotypes has been known for decades, and the current population-based analysis supports this conclusion. Note, however, that the estimated cumulative risk of 4.3% associated with the DR3/DR4 heterozygote is much lower than what has been reported in UK families (approximately 14.4%, pooling DR3/DR4 subtypes) [23]. The failure of Pitkäniemi et al. [24], when analyzing the same data, to identify anything else than DR3 and DR4 alleles to be statistically significant contributors to the risk is likely due to the ascertainment correction and the consequent substantial loss of data. Considering haplotypes, we found the same T1D associated haplotype A2/B22/DR4 (probability 0.959 of being T1D free to the age of 15) that has been identified earlier both by Tuomilehto-Wolf et al. [14] and by Thomas et al. [12], along with several more rare haplotypes with an even higher risk of T1D.

While gaining statistical power by incorporating the ascertainment process in the full likelihood, this study has some clear limitations. In terms of the analysis of T1D risk associated with HLA genotypes, genotypes DRB1*04 subtypes were not available. This is a limitation, because it is known to be the high risk genotype and would have made the analysis population based registry data more interesting. In the present study, we have not included any non-genetic measured covariate effects in the model, since there were no measurements available for the large reference data. However, in the presence of such information, the hazard model can be easily modified also to take such information into account. In principle, the risk contribution of each allele at a particular locus could be mediated through a latent partition of the haplotypes into several risk categories, in a similar manner as was done by Seaman et al. [25]. However, with the current computing power available in single workstations, such an approach would be very tedious to pursue in a numerically stable manner. Also, it would be possible to refine our approach further by modeling the familial structures in the general population, and then carry out the corresponding MCMC simulations. This would increase the computational burden significantly, and very likely lead to no real changes in the risk estimates. For the same reason, we did not explicitly account in our modeling for the uncertainties in the reconstruction of haplotypes from the BMDR data [26], although such measures would have been available from the output of the PHASE software.

We conclude that the likelihood-based Bayesian approach considered here offers a flexible and coherent framework for handling the uncertainty related to the risks associated with various types of molecular and other factors. The same conclusion has been reached recently within other areas of genetics [27], as well as applied sciences in general.

## Supporting Information

Table S1 The number of the ascertained subjects and families in the DiMe Study according to the year of diagnosis of the proband during the recruitment period from January 1, 1987 to April 30, 1989.
(0.05 MB DOC)
