# Supplementary material for: Full Likelihood Analysis of Genetic Risk with Variable Age at Onset Disease—Combining Population-Based Registry Data and Demographic Information
Source: PLoS One. 2009 Aug 31;4(8):e6836. doi: 10.1371/journal.pone.0006836 (PMC2730012; doi:10.1371/journal.pone.0006836)
Supplement: Table S1 — The number of the ascertained subjects and families in the DiMe Study according to the year of diagnosis of the proband during the recruitment period from January 1, 1987 to April 30, 1989. (0.05 MB DOC) [file pone.0006836.s001.doc]

| Number of subjects according to the year of diagnosis of the proband and the relationship with the proband |  |  |  |  |
| --- | --- | --- | --- | --- |
| Year of the diagnosis of the proband | 1987 | 1988 | 1989 | Total |
|  |  |  |  |  |
| Father | 328 | 314 | 126 | 768 |
| Mother | 328 | 314 | 126 | 768 |
| Proband | 332 | 315 | 127 | 774 |
| Siblings of the proband | 479 | 469 | 222 | 1170 |
| Total | 1467 | 1412 | 601 | 3480 |
|  |  |  |  |  |
| Number of HLA typed subjects according to the year of diagnosis of the proband and the relationship with the proband |  |  |  |  |
| Year of the diagnosis of the proband | 1987 | 1988 | 1989 | Total |
|  |  |  |  |  |
| Father | 222 | 252 | 98 | 572 |
| Mother | 240 | 280 | 110 | 630 |
| Proband | 265 | 299 | 120 | 684 |
| Siblings of the proband | 237 | 293 | 128 | 658 |
| Total | 964 | 1124 | 456 | 2544 |
|  |  |  |  |  |
| Number of families according to HLA typing available from the family members |  |  |  |  |
| Year of the diagnosis of the proband | 1987 | 1988 | 1989 | Total |
|  |  |  |  |  |
| None typed | 56 | 11 | 3 | 70 |
| Both parents typed | 71 | 79 | 37 | 187 |
| At least one parent missing | 70 | 63 | 32 | 165 |
| All subjects typed | 131 | 161 | 54 | 346 |
| Total | 328 | 314 | 126 | 768 |
